# Metformin Alleviates Steatohepatitis in Diet-Induced Obese Mice in a SIRT1-Dependent Way

**DOI:** 10.3389/fphar.2021.704112

**Published:** 2021-08-18

**Authors:** Wan-rong Guo, Juan Liu, Li-dan Cheng, Zi-yu Liu, Xiao-bin Zheng, Hua Liang, Fen Xu

**Affiliations:** ^1^ Department of Endocrinology and Metabolism, the Third Affiliated Hospital of Sun Yat-sen University, Guangzhou, China; ^2^ Guangdong Provincial Key Laboratory of Diabetology, Guangzhou, China; ^3^ Department of Endocrinology and Metabolism, the First Affiliated Hospital of Sun Yat-sen University, Guangzhou, Guangdong, China; ^4^ Department of Endocrinology and Metabolism, the Second Affiliated Hospital of Nanchang University, Nanchang, China; ^5^ Department of Gastroenterology, Shantou Central Hospital, Shantou, Guangdong, China

**Keywords:** meformin, SIRT1, NAFLD, M1 polarization, cholesterol catabolism

## Abstract

Metformin is the first-line anti-diabetic drug for type 2 diabetes. It has been found to significantly reduce liver aminotransferase in nonalcoholic fatty liver disease (NAFLD). However, whether metformin improves NAFLD progression remains controversial. Sirtuin 1 (SIRT1), an NAD^+^-dependent deacetylase, plays a vital role in hepatic steatosis and inflammation. Here, we investigated the effect of metformin on steatohepatitis and the role of SIRT1 in diet-induced obese (DIO) mice. The results showed that metformin significantly reduced body weight and fat mass of DIO mice. In addition, metformin also alleviated adiposity and hepatic steatosis, and greatly upregulated uncoupling protein 1 (UCP1) expression in adipose tissues of DIO mice. Unexpectedly, the effects of metformin on reducing body weight and alleviating hepatic steatosis were not impaired in *Sirt1* heterozygous knockout (*Sirt1* ^*+/−*^) mice. However, SIRT1-deficiency remarkably impaired the effects of metformin on lowering serum transaminases levels, downregulating the mRNA expression of proinflammatory factors, and increasing the protein level of hepatic Cholesterol 25-Hydroxylase (CH25H), a cholesterol hydroxylase in cholesterol catabolism. In summary, we demonstrated that metformin alleviates steatohepatitis in a SIRT1-dependent manner, and modulation of M1 polarization and cholesterol metabolism may be the underlying mechanism.

## Introduction

Nonalcoholic fatty liver disease (NAFLD), characterized by hepatic lipid accumulation over 5% of liver weight, includes a broad spectrum ranging from steatosis to non-alcoholic steatohepatitis (NASH), to fibrosis and cirrhosis. The global prevalence of NAFLD has been estimated to be approximately 23.75–30.45% in general population (Younossi et al., 2019). The pathogenesis of NAFLD remains a topic of discussion. Several studies suggested a bidirectional link between NAFLD and type 2 diabetes (T2D). Insulin resistance is considered to be a key factor in this connection (Anstee et al., 2013). Currently, there are no effective FDA-approved drugs for NAFLD. Due to the common pathophysiological features of NAFLD and T2D, antidiabetic drugs such as insulin sensitizers have been evaluated in patients with NAFLD. Metformin is one of these drugs (Sumida and Yoneda, 2018).

Although metformin has been used for over 60 years, its mechanism of action remains to be fully elucidated. As a first-line antidiabetic medication, metformin lowers blood glucose by decreasing gluconeogenesis in the liver, stimulates glucose intake in muscles, and increases fatty oxidation in adipose tissues (Stumvoll et al., 1995). At molecular level, metformin activates adenosine monophosphate-activated protein kinase (AMPK), a vital regulatory factor of energy metabolism (Zhou et al., 2001) (Chen et al., 2017; Rena et al., 2017), which inactivates acetyl-CoA carboxylase (ACC) and 3-hydroxy-3-methylglutaryl (HMG)-CoA reductase, and decreases fatty acid synthase (FAS) expression (Kohjima et al., 2008) (Li et al., 2011), which plays an important role in the mechanism of metformin. However, AMPK was later proven to be dispensable in the mechanism of action of metformin (Foretz et al., 2010). The beneficial effects of metformin on reducing body weight and lipid synthesis (Lin et al., 2000)^,^ (Tang et al., 2016) make it a potential medication for fatty liver. Several clinical trials have also revealed that it lowered aminotransferases and alleviated necroinflammation in patients with NAFLD (Marchesini et al., 2001; Uygun et al., 2004; Bugianesi et al., 2005). Hence, exploring the underlying mechanism will be helpful for better clinical application.

SIRT1, a member of the sirtuin family, is an NAD^+^-dependent deacetylase. It has been reported to play an important role in maintaining normal functions of brown adipose tissue (BAT) (Xu et al., 2016a) inducing beiging of white adipose tissue (Xu et al., 2016b; Baskaran et al., 2016), protecting cardiomyocytes from obesity-induced cardiac remodeling (Wang et al., 2018), and suppressing inflammation in vessels (Zhang et al., 2018). Previous studies have also shown that SIRT1 heterozygous deficient mice exhibited exacerbated HFD-induced hepatic steatosis and inflammation as well as insulin resistance (Xu et al., 2016b; Xu et al., 2010). These results indicate that SIRT1 plays an important role in energy and lipid homeostasis. Moreover, SIRT1 is also involved in the effects of fasting and exenatide (a GLP-1 receptor agonist) on NAFLD (Li et al., 2014; Xu et al., 2014). Metformin upregulates the expression of hepatic SIRT1 *in vivo* and *in vitro* (Caton et al., 2010) (Zheng et al., 2012). However, whether SIRT1 is indispensable for the effect of metformin on improving hepatic steatosis and inflammation remains unclear. Therefore, this study aimed to investigate the role of SIRT1 in the metformin-improved fatty liver and the underlying mechanisms.

## Materials and Methods

### Animal Study

Seven-week-old male C57BL/6J mice were purchased from the GemPharmatech Company (Nanjing, China). The *Sirt1* heterozygous knockout (*Sirt1* ^*+/−*^) mice with a C57BL/6J genetic background were obtained from the Pennington Biomedical Research Center (Louisiana State University, Baton Rouge, LA, United States) and bred in the animal center of the Third Affiliated Hospital of Sun Yat-sen University. SIRT1 protein levels in the liver of *Sirt1* ^*+/−*^ mice were detected and the expression of SIRT1 was found impaired (Xu et al., 2014) (Zheng et al., 2017).

Mice were housed at 22 ± 2°C and 50 ± 5% of humidity with free access to water and food under a 12-h light/dark cycle in a standard specific-pathogen-free environment. After a 1-week acclimation period, mice were divided into two groups randomly fed with a chow diet (Guangdong Medical Laboratory Animal Center, Guangzhou, China) or a high-fat diet (HFD) (D12331, Research Diets, United States). To establish an NAFLD animal model, mice were fed with an HFD for 12 weeks (Van Herck et al., 2017). After a 12-weeks dietary intervention, mice fed with different diets were randomly divided into two groups with free access to drinking water with or without metformin (S1950, Selleck, TX, United States) for 4 weeks. The dose of metformin was 200–250 mg/kg/d, which was equivalent to a human metformin dose of 20 mg/kg/d orally (Foretz et al., 2014). Body weight, fasting blood glucose (FBG), and food intake was recorded throughout the study. The intraperitoneal glucose tolerance test (i.p.GTT) and insulin tolerance test (i.p.ITT) were performed on all mice during the last week of the metformin treatment period. Finally, the mice were sacrificed by cervical dislocation after isoflurane inhalation anesthesia to collect blood samples, liver, and adipose tissues. All tissues were collected and kept in liquid nitrogen immediately, then stored at −80°C. There were four to five mice in each group.

The animal experiment complied with the ARRIVE guidelines and the National Institutes of Health Guide for the Care and Use of Laboratory Animals. All protocols were approved by Sun Yat-sen University Institutional Animal Care and Use Committee.

### Intraperitoneal Glucose Tolerance Test and Insulin Tolerance Test

Mice were fasted overnight before the i.p.GTT and administered intraperitoneally with glucose (2.0 g/kg wt). For the i.p.ITT, mice were injected intraperitoneally with insulin (0.65 units/kg wt) after fasting for 4 h. Baseline blood glucose (0 min) and blood glucose at 30, 60, and 120 min after the injection were recorded during the i.p.GTT and i.p.ITT.

### RT-qPCR

Total RNA was isolated from tissues using Tri Reagent (Sigma, MO, United States) according to the manufacturer’s instructions. Reverse transcription (RT) of RNA was performed with a Transcriptor First Strand cDNA Synthesis Kit (Roche Applied Science, Switzerland). Real-time PCR was performed using a LightCycler 480 System with LightCycler 480 SYBR Green Master (Roche Applied Science, Switzerland). For each gene, mRNA expression was calculated relative to that of β-actin, and the fold change in mRNA expression was determined using the 2^−ΔΔCT^ method.

### Western Blotting

Total protein was extracted from frozen tissues using RIPA lysis and extraction buffer (89,900, Thermo Fisher, MA, United States) with Halt™ protease and phosphatase Inhibitor Cocktail (78,440, Thermo Fisher, MA, United States). Equal amounts of protein were separated by 10% SDS-PAGE and blotted onto a polyvinylidene fluoride membrane (Millipore, MA, United States). Membranes were incubated with primary antibodies at 4°C overnight and then with secondary antibodies (1:10,000, LICOR IRDye 800CW) for 1 h, and the imaged was taken using the Odyssey Infrared Imaging System. Antibodies against ACC (3676S), FAS (3180S), SCD1 (2794S), SIRT1 (9475S) and β-actin (4970S) were purchased from Cell Signaling Technology (Danvers, MA, United States). Antibody against CH25H (sc-293256) was purchased from Santa Cruz (Texas, United States).

### Quantification of Triglyceride and Total Cholesterol in Liver

The triglyceride and cholesterol contents in the liver were measured using a triglyceride quantification colorimetric kit (K622-100, Biovision, CA, United States) and a cholesterol quantification colorimetric kit (K603-100, Biovision, CA, United States) according to the manufacturer’s protocols. Serum alanine transaminase (ALT) and aspartate transaminase (AST) were detected using an ALT quantification kit (E-BC-K235-M, Elabscience, Wuhan, China) and an AST quantification kit (E-BC-K236-M, Elabscience, Wuhan, China) according to the manufacturer’s protocols.

### Hematoxylin and Eosin (HE) Staining

Fresh tissues were collected in 4% paraformaldehyde solution (GBCBIO, Guangzhou, China) overnight. Then, the tissues were embedded in paraffin and sectioned at 3–5 μm at room temperature. After paraffinizing with fresh xylene twice and rehydrating with ethanol at a gradient concentration, the nucleus was stained with hematoxylin, and the cytoplasm was stained with eosin. Photomicrographs were taken under a DMi8 inverted microscope (Leica Microsystems, Wetzlar, Germany) at ×200 magnification.

### Oil Red O Staining

Fresh liver tissues were embedded in OCT compounds (Sakura, United States) and stored at −80°C. The slides were obtained through serial cross-section cutting at a thickness of 6–8 μm at −18°C. A stock solution of 0.5% Oil Red O was prepared in isopropanol, and the working concentration of 60% was diluted with ddH2O. After washing with 60% isopropanol, the prepared sections were stained with Oil Red O working solution for 1 h and washed with running tap water for 20 min. The nuclei were then stained with hematoxylin for 1 min. The whole procedure was protected from light and the photomicrographs were taken in 2 h.

### Immunohistochemistry

The tissues in paraffin were sectioned at 3–5 μm. After deparaffinization and dehydration described previously, antigen retrieval was performed with heated citrate buffer for 5 min and washed with PBS three times. The sections were blocked with 3% hydrogen peroxidase for 10 min and washed with PBS three times again. The sections were then incubated with primary antibody overnight at 4°C and then with a secondary antibody for 1 h at 37°C. A DAB chromogenic solution was used to detect the reaction reagents and hematoxylin was used to stain the nuclei. Image-Pro Plus 6.0 was used to analyze the protein expression. Antibody against UCP1 (Ab10983) was purchased from Abcam (Cambridge, United Kingdom). Antibody against F4/80 (sc-71085) was purchased from Santa Cruz (Texas, United States). Antibodies against iNOS (GB11119) and CD206 (GB13438) were purchased from Service-bio (Wuhan, China).

### Statistical Analysis

Data are presented as mean ± SEM. Differences between groups were evaluated by two-way ANOVA. Statistical significance was set at P < 0.05.

## Results

### Metformin Improves Obesity and Glucose Metabolic Disorders in DIO Mice

**FIGURE 1:**
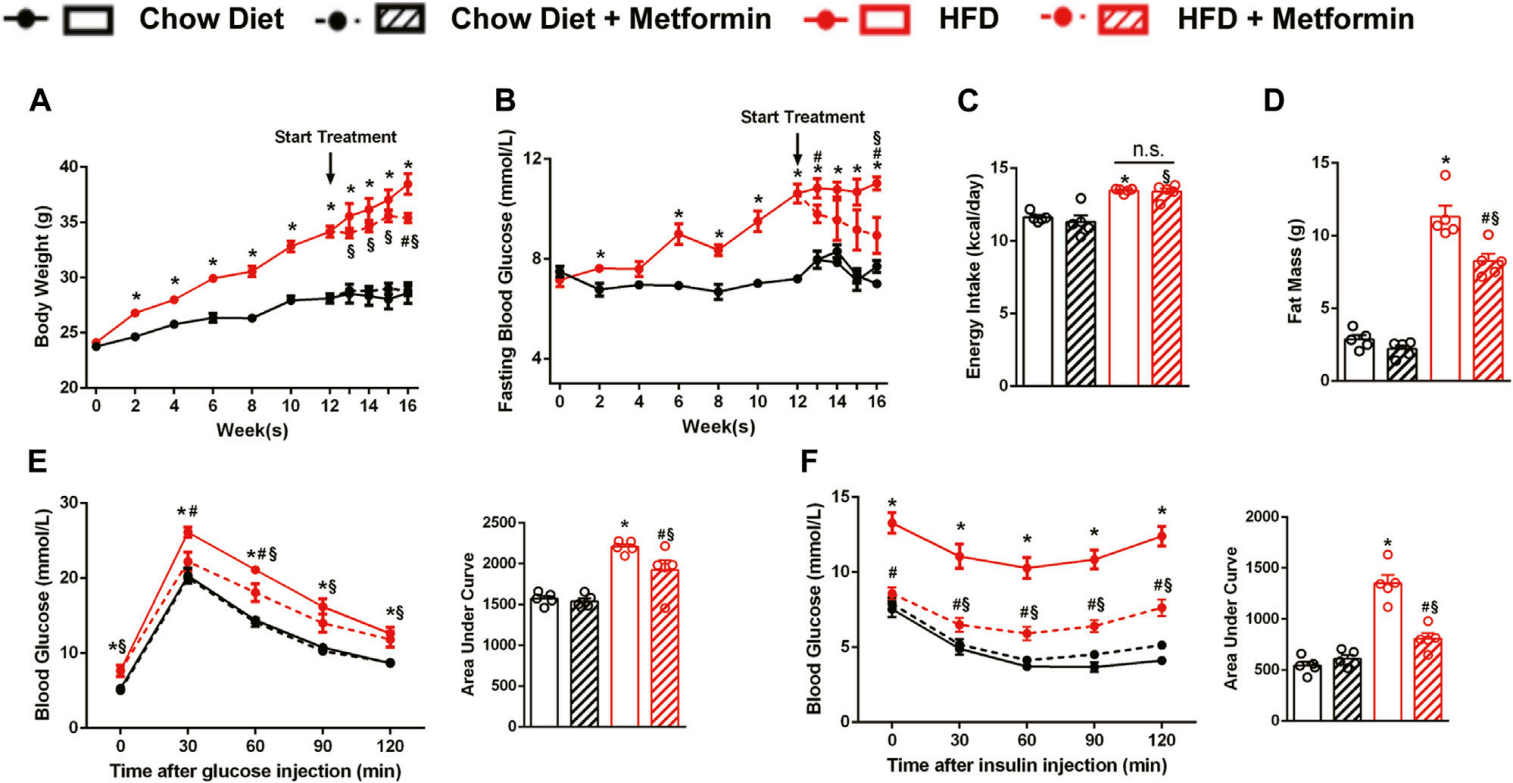
Metformin improves obesity and glucose metabolic disorders in DIO mice. C57BL/6J mice were treated with either metformin or drinking water as control for 4 weeks after a 12-weeks diet intervention. In DIO mice, metformin significantly reduced **(A)** body weight (*p* < 0.05), **(B)** fasting blood glucose (*p* < 0.05) but showed no remarkable effect on **(C)** energy intake (*p* > 0.05). **(D)** Body fat mass of DIO mice was greatly reduced by metformin (*p* < 0.05). **(E)** The Intraperitoneal glucose tolerance test and **(F)** intraperitoneal insulin tolerance test showed that metformin significantly improved glucose tolerance and insulin sensitivity (*p* < 0.05). Data are shown as mean ± SEM. *p < 0.05, HFD group vs. Chow diet group; ^#^ *p* < 0.05, HFD group vs. HFD + Metformin group; ^§^ *p* < 0.05, HFD + Metformin vs. Chow diet group.

After the diet intervention, body weight and FBG of DIO mice were higher than those of the control mice fed with a chow diet. After a 4-week treatment, metformin reduced the weight and improved FBG levels in DIO mice (Figures 1A,B). However, the result of energy intake calculated by food intake detected every 2 weeks did not show a reduction with metformin treatment (Figure 1C). Consistent with body weight, fat mass of mice treated with metformin decreased (Figure 1D). Moreover, metformin improved glucose tolerance and insulin sensitivity in DIO mice (Figures 1E,F). These results demonstrate that metformin improves obesity and obesity-related metabolic disorders in DIO mice.

### Metformin Induces Beiging of White Adipose Tissue and Activation of brown Adipose Tissue

**FIGURE 2:**
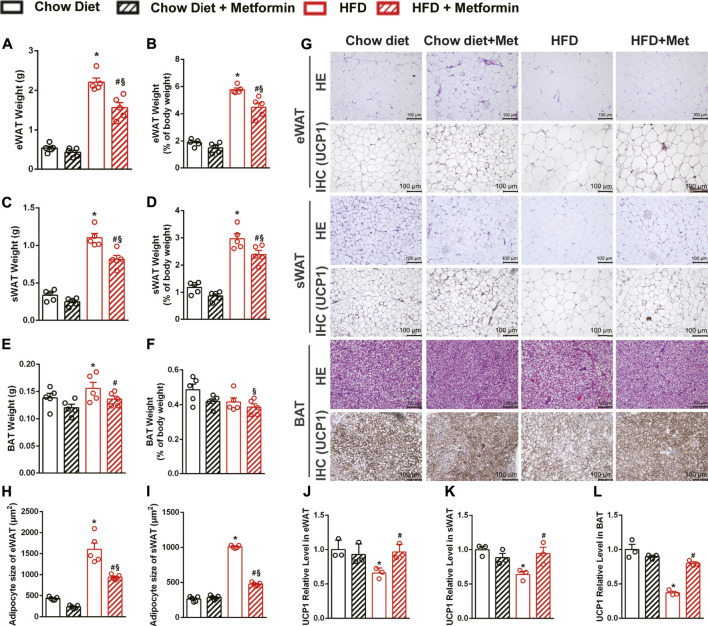
Metformin induces beiging of WAT and activation of BAT in DIO mice. In DIO mice, the weight of **(A)** epididymal adipose tissue (eWAT), **(C)** subcutaneous adipose tissue (sWAT), **(E)** brown adipose tissue (BAT) were significantly reduced by metformin (*p* < 0.05). Metformin also reduced the percent of **(B)** eWAT and **(D)** sWAT to body weight (*p* < 0.05) while had no effect on the percent of **(F)** BAT (*p* > 0.05). **(G)** The H&E staining and the uncoupling protein 1 (UCP1) immunohistochemical staining of adipose tissue with x200 magnification showed that metformin induced beiging of WAT and activation of BAT, which were confirmed by the results of quantification of UCP1 protein expressions in **(J)** eWAT, **(K)** sWAT, and **(L)** BAT (*p* < 0.05). The size of adipocytes was significantly reduced in **(H)** eWAT and **(I)** sWAT by metformin (*p* < 0.05). Data are shown as mean ± SEM. *p < 0.05, HFD group vs Chow diet group; ^#^ *p* < 0.05, HFD group vs. HFD + Metformin group; ^§^ *p* < 0.05, HFD + Metformin vs. Chow diet group.

To investigate how metformin reduces body weight and fat mass, adipose tissues were collected and weighed. The weight of epididymal white adipose tissue (eWAT), subcutaneous white adipose tissue (sWAT), and BAT of mice fed with an HFD were decreased by metformin (Figures 2A,C,E). The precent of eWAT and sWAT to body weight were decreased as well by metformin on HFD diet (Figures 2B,D). The H&E staining also revealed that metformin decreased the size of adipocytes in adipose tissues including eWAT, sWAT, and BAT (Figures 2G–I). Immunohistochemical staining of UCP1, which is a marker of WAT beiging, revealed that the expression of UCP1 downregulated by HFD recovered after 4-weeks metformin treatment in eWAT (Figures 2G,J), sWAT (Figures 2G,K), and BAT (Figures 2G,L). SIRT1 expression was significantly suppressed by HFD-feeding in those adipose tissues (Supplementary Figure S1). These data demonstrate that metformin ameliorated the adiposity in DIO mice through beiging of WAT and activation of BAT, as indicated by the recovered expression of UCP1 in adipose tissues.

### Metformin Alleviates Hepatic Steatosis in DIO Mice

**FIGURE 3:**
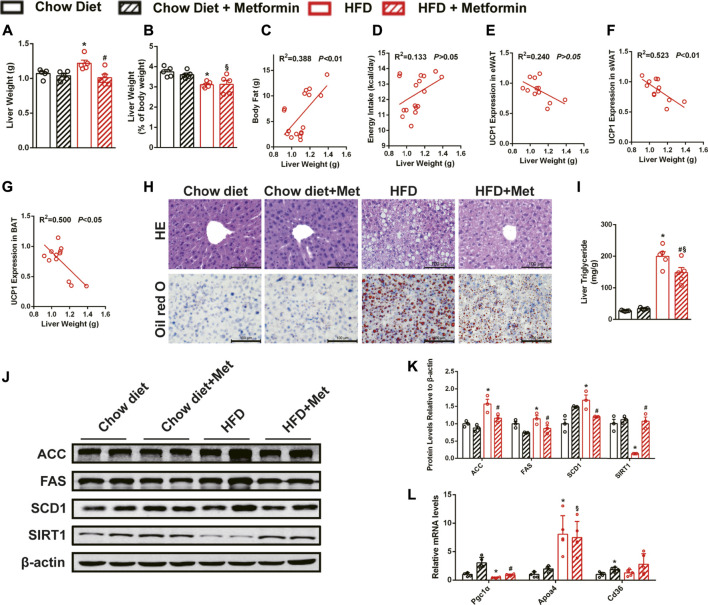
Metformin alleviates hepatic steatosis in DIO mice. Metformin reduced **(A)** liver weight and did not change **(B)** the liver weight to body weight ratio in DIO mice. **(C)** Body fat, UCP1 expressions in **(F)** sWAT and **(G)** BAT but not **(D)** energy intake or **(E)** UCP1 expression in eWAT were correlated with liver weight. **(H)** The H&E staining, the Oil Red O staining of the liver with ×400 magnification, and **(I)** liver triglyceride content indicated that metformin alleviated hepatic steatosis (*p* < 0.05). Metformin downregulated **(J,K)** the proteins expressions related to lipid metabolism (*p* < 0.05) and **(L)** the mRNA expressions of lipid metabolism genes in the liver (*p* < 0.05). Data are shown as mean ± SEM. *p < 0.05, HFD group vs. Chow diet group; ^#^ *p* < 0.05, HFD group vs. HFD + Metformin group; ^§^ *p* < 0.05, HFD + Metformin vs. Chow diet group.

Liver weight of DIO mice was reduced by metformin treatment (Figure 3A), and liver weight to body weight ratio was not changed (Figure 3B). Liver weight was positively correlated with body fat (Figure 3C) and negatively correlated with UCP1 expressions in sWAT (Figure 3F) and BAT (Figure 3G). No correlation was observed between liver weight and energy intake (Figure 3D) or UCP1 expression in eWAT (Figure 3E). The histological results, including the HE staining and Oil Red O staining, showed that metformin alleviated HFD-induced hepatic steatosis (Figure 3H). Furthermore, the quantification of lipids in the liver also showed that metformin reduced triglyceride (Figure 3I). Metformin also reduced the protein levels of enzymes related to ACC, FAS, and stearoyl-CoA desaturase 1 (SCD1), and significantly upregulated the SIRT1 expression in the liver of DIO mice (Figures 3J,K). The mRNA level of *Pgc1α* was significantly increased in the metformin group (Figure 3L). These results confirm that metformin ameliorated hepatic steatosis in DIO mice and SIRT1 may be one of the targets in the metformin-alleviated fatty liver.

### The Effect of Metformin in Alleviating Hepatic Steatosis Remained in SIRT1 Deficient Mice

**FIGURE 4:**
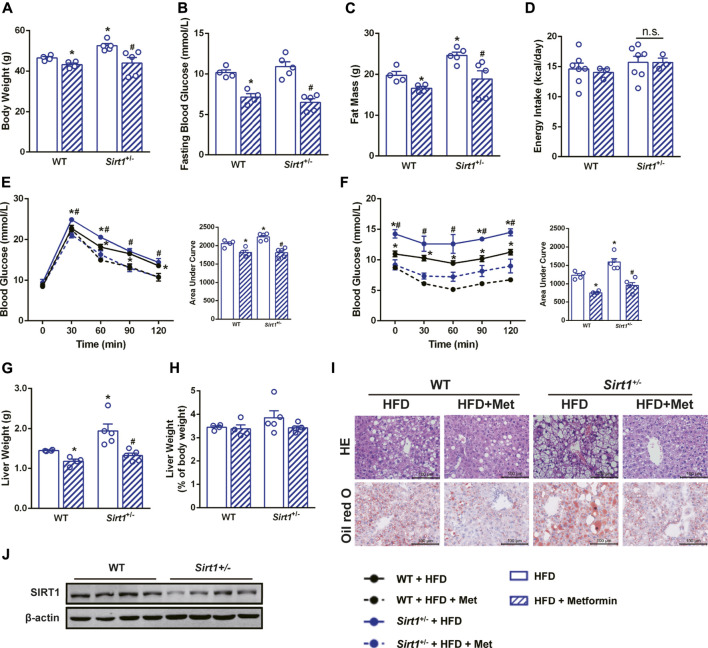
The effect of metformin on reducing body weight and alleviating hepatic steatosis remains in SIRT1 deficient mice. SIRT1 deficiency did not impair the beneficial effects of metformin in **(A)** body weight, **(B)** fasting blood glucose and **(C)** body fat mass (*p* < 0.05). **(D)** Energy intake remained unaffected in *Sirt1* ^*+/−*^ mice (*p* > 0.05). Metformin improved **(E)** glucose tolerance and **(F)** insulin sensitivity in *Sirt1* ^+/−^ mice. **(G)** Liver weight, **(H)** the liver weight to body weight ratio, **(I)** the H&E staining, and the Oil Red O staining with ×400 magnification also showed that metformin alleviated hepatic steatosis in *Sirt1* ^*+/−*^ mice (*p* < 0.05). **(J)** SIRT1 protein expression was lower in liver of *Sirt1* ^+/−^ mice. Data are shown as mean ± SEM. *p < 0.05 WT + HFD + Metformin or *Sirt1* ^*+/−*^ + HFD vs. WT + HFD group; ^#^ *p* < 0.05, *Sirt1* ^*+/−*^ + HFD + Metformin vs. Sirt1*1* ^*+/−*^ + HFD group.

To further clarify the role of SIRT1 in the effect of metformin on the liver, *Sirt1* ^*+/−*^ mice and their wild-type (WT) littermates were also treated with metformin after a 12-weeks HFD challenge. Unexpectedly, metformin still reduced body weight, FBG, and body fat, improved glucose tolerance as well as insulin sensitivity of *Sirt1* ^*+/−*^ mice (Figures 4A–C,E,F), and did not have any effect on their energy intake (Figure 4D). Moreover, metformin reduced liver weight (Figure 4G) without changing the liver weight to body weight ratio (Figure 4H) and ameliorated the hepatic steatosis in *Sirt1* ^*+/−*^ mice (Figure 4I). SIRT1 protein level in liver of *Sirt1* ^+/−^ mice was confirmed to be significantly lower than that in WT mice (Figure 4J). These results suggest that SIRT1 heterozygous deficiency does not impair the effect of metformin on body weight, FBG, and hepatic steatosis.

### The Effect of Metformin on Ameliorating Diet-Induced Steatohepatitis Is Diminished in SIRT1 Deficient Mice

**FIGURE 5:**
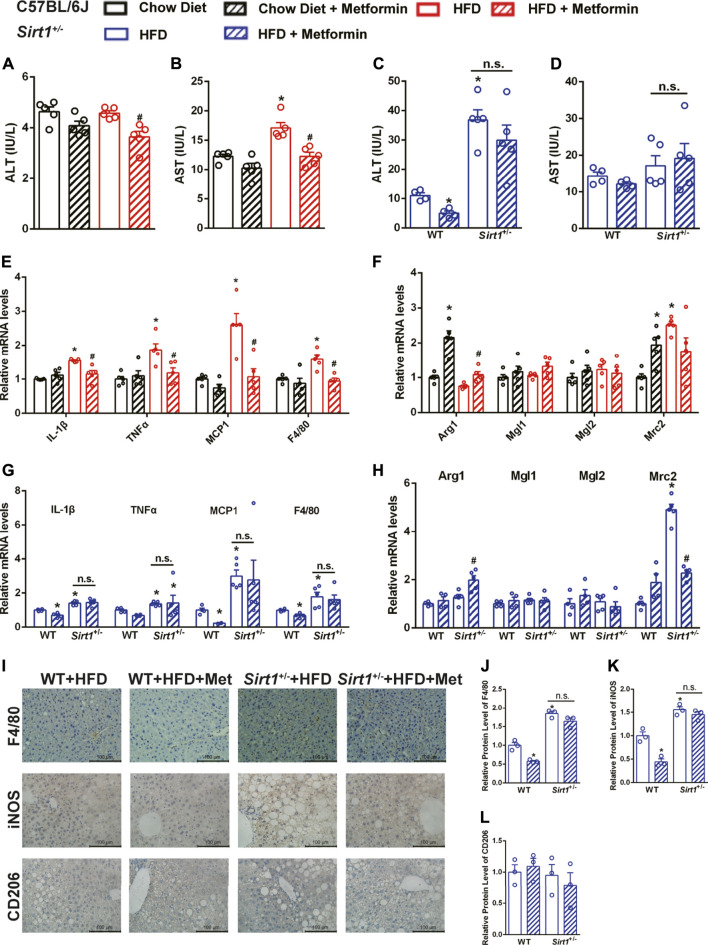
The effect of metformin on ameliorating diet-induced steatohepatitis is diminished in *Sirt 1* ^*+/-*^ mice. After metformin treatment, **(A)** Serum alanine transaminase (ALT), **(B)** Aspartate transaminase (AST) and **(E)** the relative mRNA levels of M1 markers were significantly decreased (*p* < 0.05) while **(F)** the relative mRNA levels of M2 markers were unaffected in DIO mice (*p* > 0.05). SIRT1 deficiency impaired the effect of metformin on decreasing **(C)** serum ALT levels and downregulating **(G)** the relative mRNA expressions of M1 markers (*p* > 0.05). **(H)** The relative mRNA levels of M2 markers were also unaffected by metformin in Sirt1*1* ^*+/−*^ mice (*p* > 0.05). **(I–L)** The F4/80, M1 markers iNOS, M2 markers CD206 immunohistochemical staining, and the quantification of those protein expressions of the liver in *Sirt11* ^*+/−*^ mice also confirmed that the metformin-induced downregulation of pro-inflammatory factors was impaired in *Sirt1* ^*+/−*^ mice (*p* > 0.05). Data are shown as mean ± SEM. In C57BL/6 mice, *p < 0.05, HFD group vs Chow diet group; ^#^ *p* < 0.05, HFD group vs HFD + Metformin group. In *Sirt11* ^*+/−*^ mice, *p < 0.05, WT + HFD + Metformin or *Sirt1* ^*+/−*^ + HFD vs. WT + HFD group, ^#^ *p* < 0.05, *Sirt1* ^*+/−*^ + HFD + Metformin vs. *Sirt1* ^*+/−*^ + HFD group.

In DIO mice, metformin decreased both serum ALT and AST levels (Figures 5A,B) and decreased the mRNA expression of M1 macrophage markers including interleukin-1β (IL-1β) and tumor necrosis factor-alpha (TNFα), which are also pro-inflammatory markers (Figure 5E). The M2 macrophage markers, Arg1 was upregulated, while Mgl1, Mgl2, and Mrc2 were unaffected in the liver of DIO mice after metformin treatment (Figure 5F), it indicated that metformin might promote the polarization of macrophages toward M2 macrophages. However, both the serum ALT levels and the mRNA expression of M1 macrophage markers were higher in *Sirt1* ^+/−^ mice than in WT mice on HFD, and metformin neither decreased serum ALT levels (Figure 5C) nor downregulated the elevated expression of M1 macrophage markers in *Sirt1* ^+/−^ mice (Figure 5G). M2 macrophage markers seemed not affected by metformin in *Sirt1* ^+/−^ mice (Figure 5H). The protein expression of F4/80, a macrophage marker in the liver, further illustrated that SIRT1 deficiency diminished the protective effect of metformin on hepatic inflammation (Figures 5I,J). The results of protein expression of iNOS (an M1 macrophage marker), and CD206 (an M2 macrophage marker) confirmed that SIRT1 deficiency diminished the metformin-induced suppression of the M1 polarization (Figures 5I,K,L). Taken together, metformin alleviates hepatic inflammation by suppressing the M1 macrophage polarization in a SIRT1-dependent way.

### SIRT1 Deficiency Affects the Effect of Metformin on Regulating Cholesterol Metabolism

In addition to modulating macrophage polarization, metformin has also been reported to promote synthesis of bile acids which are catabolized from cholesterol (Sun et al., 2018). Cholesterol accumulation is crucial for the progression of the fatty liver to NASH (Wang et al., 2020). Therefore, we investigated whether metformin promoted cholesterol catabolism to alleviate hepatic inflammation.

**FIGURE 6:**
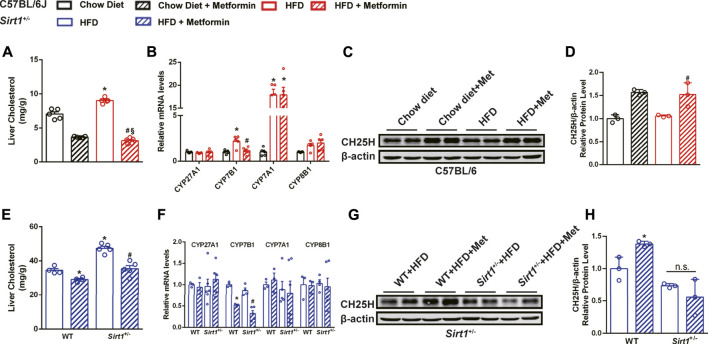
SIRT1 deficiency impairs the effect of metformin on regulating cholesterol catabolism. Metformin reduced **(A)** hepatic cholesterol content in DIO mice (*p* < 0.05). **(B)** The relative mRNA level of CYP7B1 in the liver of DIO mice was reduced by metformin (*p* < 0.05). **(C,D)** The relative protein expression of CH25H was significantly upregulated (*p* < 0.05) but that of CYP7B1 was unaffected (*p* > 0.05) by metformin in the liver of DIO mice. Although **(E)** metformin also reduced hepatic cholesterol content (*p* < 0.05) and **(F)** downregulated the mRNA expression of CYP7B1 in *Sirt1* ^*+/−*^ mice (*p* < 0.05), **(G,H)** the protein levels of CH25H were unaffected by metformin in the liver of *Sirt11* ^*+/−*^ mice (*p* > 0.05). In C57BL/6J mice, *p < 0.05, HFD group vs Chow diet group, ^#^ *p* < 0.05, HFD + Metformin vs. HFD group. In *Sirt1* ^*+/−*^ mice, *p < 0.05, WT + HFD + Metformin or *Sirt1* ^*+/−*^ + HFD vs. WT + HFD group, ^#^ *p* < 0.05, *Sirt1* ^*+/−*^ + HFD + Metformin vs. *Sirt1* ^*+/−*^ + HFD group.

In DIO mice, there was a significant decrease in the liver cholesterol content after metformin treatment (Figure 6A). To confirm how metformin promotes cholesterol catabolism, several genes, including CYP27A1, CYP7B1, CYP7A1, and CYP8B1 related to cholesterol catabolism were tested by qPCR. The results showed that the mRNA expression of CYP7B1 was significantly downregulated by metformin (Figure 6B). We further found that the protein level of CH25H, a cholesterol hydroxylase, was upregulated by metformin in the liver of DIO mice (Figures 6C,D). Under HFD feeding, metformin also reduced the cholesterol content in the livers of WT mice and *Sirt1* ^*+/−*^ mice (Figure 6E). The mRNA expression of CYP7B1 was also downregulated by metformin in *Sirt1* ^*+/−*^ mice (Figure 6F). Nevertheless, metformin upregulated CH25H in the liver of WT mice but did not exert this effect in *Sirt1* ^*+/−*^ mice (Figures 6G,H). These data suggest that metformin promotes cholesterol catabolism mainly by activating CH25H in a SIRT1-dependent manner, which could be one of the underlying mechanisms of metformin-improved steatohepatitis.

## Discussion

Although clinical evidence demonstrates that metformin does not improve the histology of fatty liver in NAFLD patients, we observed metformin-alleviated fatty liver in the DIO mouse model. This is consistent with what was observed in other obese mouse models (Song et al., 2015; Tang et al., 2016). Moreover, metformin has been reported to lower hepatic transaminases which are markers of hepatitis (Iogna Prat and Tsochatzis, 2018). In this study, we also confirmed that metformin ameliorated HFD-induced steatohepatitis, indicated by the decrease in serum ALT and AST levels and the suppressive effect of metformin on the expression of pro-inflammatory cytokines. As an activator of AMPK, metformin regulates lipid metabolism by suppressing the hepatic expression of lipogenic enzymes, including ACC (Fullerton et al., 2013) and SREBP1 (Li et al., 2011). Consistent with previous studies, the present study confirmed the downregulation of ACC, FAS, and SCD1 by metformin. These results confirm that metformin effectively improved hepatic steatosis.

Although AMPK and SIRT1 play critical roles in the regulation of many cellular and metabolic processes, it is difficult to determine their clear relationship because of their interplay. Moderate dose of resveratrol (a SIRT1 activator) have been reported to stimulate AMPK and improve mitochondrial function both *in vitro* and *in vivo* in a SIRT1-dependent way (Price et al., 2012). Conversely, in another study, SIRT1 was found to be downstream of AMPK(Park et al., 2012), in which resveratrol did not stimulate SIRT1 activity without functional AMPK. AMPK does not directly phosphorylate SIRT1, but its effect of this kinase on lipid oxidation is to modify the NAD^+^/NADH ratio, thus modulating SIRT1 deacetylase activity (Cantó et al., 2009). Metformin works partly through the activation of AMPK(Rena et al., 2017). However, whether the effect of metformin on improving hepatic steatosis depends on SIRT1 remains unclear. In the current study, we found that metformin still effectively alleviated HFD-induced hepatic steatosis in *Sirt1* ^+/−^ mice. The possibility for this is that SIRT1 only partly mediates metformin-improved hepatic steatosis by regulating *de novo* lipogenesis, similar to AMPK. When SIRT1 is heterozygously deficient, an alternative pathway could compensate for this effect.

Inflammation which is characterized by the presence of macrophage infiltration and increased levels of pro-inflammatory cytokines contributes to the progression of hepatic steatosis to steatohepatitis (Schuster et al., 2018;^,^ Chen et al., 2020). Macrophages play a central role in NAFLD development with pro-inflammatory macrophages (M1) determining disease severity (Kazankov et al., 2019). In experimental models, pro-inflammatory cytokines produced by M1 macrophages induce hepatic insulin resistance (Huang et al., 2010). The subpopulation paradigm of macrophages (M1/M2 paradigm) has been challenged. Some scholars believe that it is more reasonable to distinguish the subtypes of macrophages based on the stimuli they encounter and functions (Martinez and Gordon, 2014) (Nahrendorf and Swirski, 2016). In addition, dynamic observation of macrophage phenotype and function changes is also of great significance (Martinez and Gordon, 2014). In the present study, metformin significantly downregulated the pro-inflammatory cytokines including IL-1β, TNF-α, MCP-1, F4/80, and iNOS, which are markers of M1 macrophages, in DIO mice. However, SIRT1 deficiency diminished the above effects of metformin. We did not observe consistent evidence of the polarization of macrophages to the M2 type induced by metformin, which is an anti-inflammatory type of macrophage. The metformin-improved hepatic steatosis in *Sirt1* ^+/−^ mice may have an indirect effect on reducing hepatic inflammation. However, the hepatitis was not improved at all by metformin in *Sirt1* ^+/−^ mice, which indicates that SIRT1 deficiency promotes steatohepatitis and this effect is independent of the improved hepatic steatosis. Conclusively, the data suggest that metformin alleviated steatohepatitis by suppressing the M1 macrophage polarization in a SIRT1-dependent manner.

Metformin has been reported to decrease aspartate aminotransferase and alanine transaminase levels in NAFLD patients, markers of liver injury, indicating that metformin alleviated hepatitis of NAFLD patients (Bugianesi et al., 2005; Zhou et al., 2018). In the zebrafish-based NAFLD model, metformin alleviated HFD-induced steatohepatitis by reducing the polarization of macrophages to pro-inflammatory M1 macrophages (de Oliveira et al., 2019). In contrast, SIRT1 is involved in the pathogenesis of the inflammation-associated fatty liver disease. Macrophage infiltration with increased macrophage marker expression in the liver, such as MCP-1, F4/80, and CD11β, was observed in liver-specific knockout or whole-body SIRT1 heterozygous knockout mice fed with an HFD (Purushotham et al., 2009; Xu et al., 2010). SIRT1 overexpression improved effects on HFD-induced fatty liver and pro-inflammatory cytokines via downregulation of NF-κB activity (Pfluger et al., 2008). To the best of our knowledge, the present study innovatively revealed the important role of SIRT1 in metformin-mediated hepatic inflammation.

Recent research has found that cholesterol metabolism is closely related to the pathogenesis and severity of NASH (Arguello et al., 2015). The over-abundance of free cholesterol in the liver induces an unfolded protein response and generates toxic oxysterol. Moreover, free cholesterol activates hepatic Kupffer and stellate cells to produce pro-inflammatory cytokines and collagen (Arguello et al., 2015). Although SIRT1-deficiency did not impair the effect of metformin on reducing liver cholesterol content which is consistent with the hepatic steatosis result, the cholesterol hydroxylase CH25H, which is involved cholesterol catabolism, was upregulated in DIO mice after metformin treatment but not in *Sirt1* ^+/−^ mice. CH25H catabolizes cholesterol to 25-HC, and the latter has been reported to inhibit inflammation mediated by IL-1β (Reboldi et al., 2014). In brief, the present study aimed to explore the role of cholesterol metabolism involved in metformin-improved steatohepatitis, and the results suggest that the effect of metformin on regulating CH25H depends on SIRT1. The SIRT1-CH25H pathway may be another mechanism of metformin-induced alleviation of hepatic inflammation. Further studies are warranted to elucidate this the phenomenon.

**FIGURE 7:**
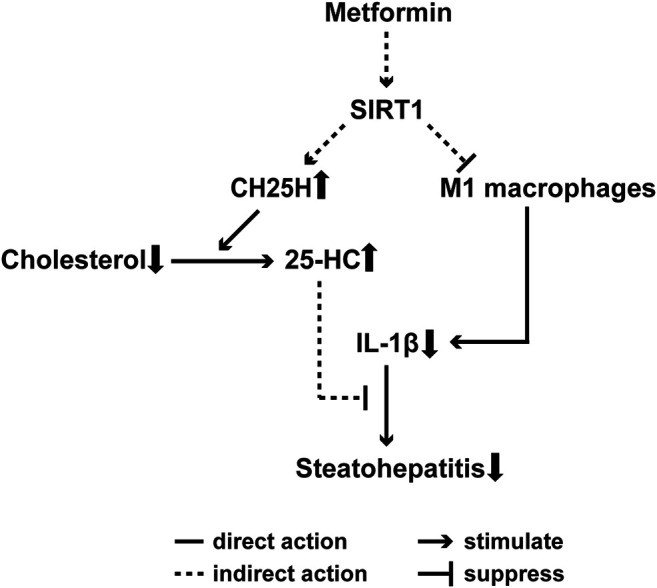
Proposed mechanism for metformin-alleviated steatohepatitis. A SIRT1-dependent cholesterol catabolism and M1 macrophages polarization are involved in metformin-induced alleviation of steatohepatitis in DIO mice. Metformin upregulates CH25H and may result in increased level of 25-HC, which has been reported to inhibit IL-1β-driven inflammation, thus ameliorates hepatic inflammation. On the other hand, metformin also downregulates the M1 macrophage markers, which suggests that metformin may inhibit the M1 macrophages polarization to alleviate steatohepatitis. Importantly, SIRT1 deficiency impairs the beneficial effects of metformin on steatohepatitis.

In summary, our study demonstrates that metformin alleviates steatohepatitis by modulating M1 macrophage polarization and regulating cholesterol catabolism in a SIRT1-dependent manner. It has been reported reports that the regulation of cholesterol hydroxylase CH25H via a SIRT1 dependent pathway may underlie the mechanism of metformin-improved steatohepatitis (Figure 7). This study is of value to elucidate the mechanism of metformin in the treatment of NAFLD and shed light on the clinical application of metformin in NAFLD patients.

## Data Availability

The raw data supporting the conclusions of this article will be made available by the authors, without undue reservation.
