# Supplementary figures and images for: Metformin Alleviates Steatohepatitis in Diet-Induced Obese Mice in a SIRT1-Dependent Way
Source: Front Pharmacol. 2021 Aug 18;12:704112. doi: 10.3389/fphar.2021.704112 (PMC8416468; doi:10.3389/fphar.2021.704112)

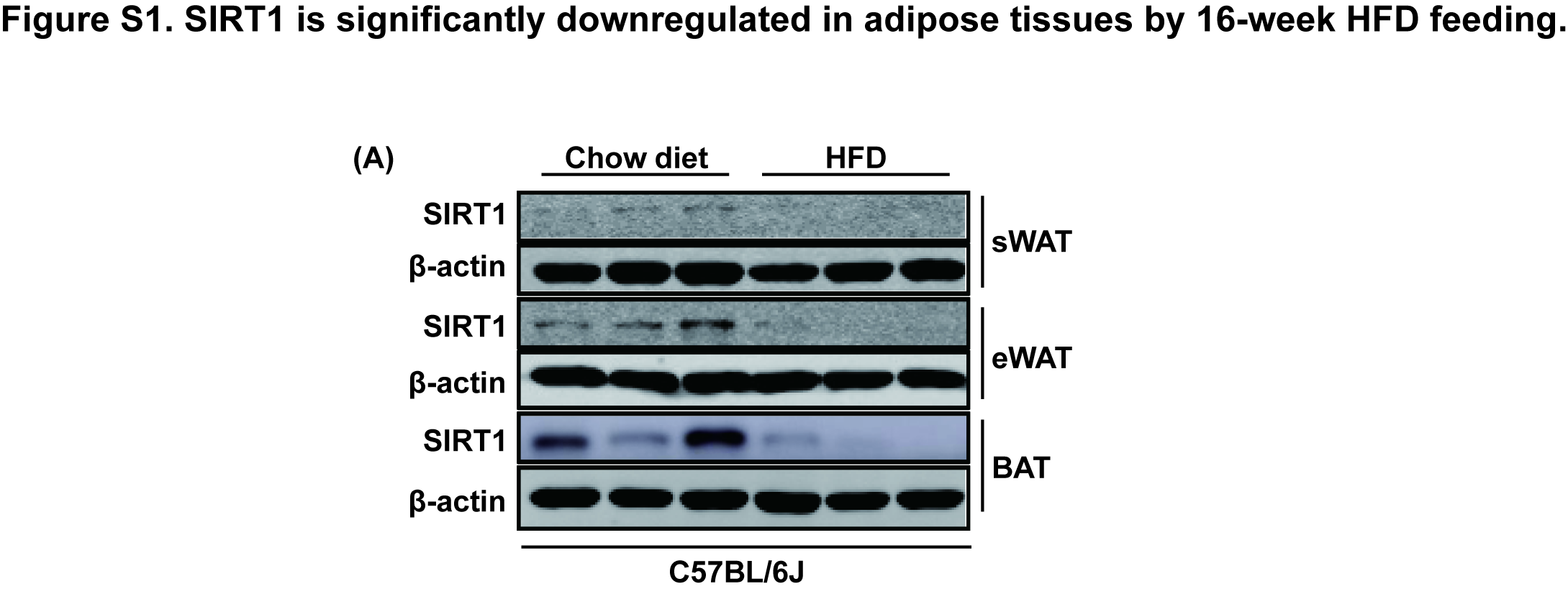

Supplement: Supplementary file 1 [file Image1.tif]
